# What determines complications and prognosis among patients subject to multivisceral resections for locally advanced gastric cancer?

**DOI:** 10.1007/s00423-023-03187-7

**Published:** 2023-11-21

**Authors:** Łukasz Bobrzyński, Radosław Pach, Antoni Szczepanik, Piotr Kołodziejczyk, Piotr Richter, Marek Sierzega

**Affiliations:** First Department of Surgery, Jagiellonian University Medical College, 2 Jakubowski Street, 30-688, Cracow, Poland

**Keywords:** Gastric cancer, Locally advanced cancers, Multivisceral resections, Splenectomy, Pancreatectomy, Colectomy, Nomogram

## Abstract

**Background:**

Locally advanced gastric cancer (GC) extending to the surrounding tissues may require a multivisceral resection (MVR) to provide the best chance of cure. However, little is known about how the extent of organ resection affects the risks and benefits of surgery.

**Methods:**

An electronic database of patients treated between 1996 and 2020 in an academic surgical centre was reviewed. MVRs were defined as partial or total gastrectomy combined with splenectomy, distal pancreatectomy, or partial colectomy.

**Results:**

Suspected intraoperative tumour invasion of perigastric organs (cT4b) was found in 298 of 1476 patients with non-metastatic GC, and 218 were subject to MVRs, including the spleen (*n* = 126), pancreas (*n* = 51), and colon (*n* = 41). MVRs were associated with higher proportions of surgical and general complications, but not mortality. A nomogram was developed to predict the risk of major postoperative morbidity (Clavien–Dindo’s grade ≥ 3a), and the highest odds ratio for major morbidity identified by logistic regression modelling was found for distal pancreatectomy (2.53, 95% CI 1.23–5.19, *P* = 0.012) and colectomy (2.29, 95% CI 1.04–5.09, *P* = 0.035). Margin-positive resections were identified by the Cox proportional hazards model as the most important risk factor for patients’ survival (hazard ratio 1.47, 95% CI 1.10–1.97). The extent of organ resection did not affect prognosis, but a MVR was the only factor reducing the risk of margin positivity (OR 0.44, 95% CI 0.21–0.87).

**Conclusions:**

The risk of multivisceral resections is associated with the organ being removed, but only MVRs increase the odds of complete tumour clearance for locally advanced gastric cancer.

**Supplementary Information:**

The online version contains supplementary material available at 10.1007/s00423-023-03187-7.

## Introduction

The primary aim of treatment for solid malignancies, including gastric cancer, is a radical surgical resection (R0), i.e. complete macroscopic and microscopic removal of the tumour [1, 2]. Since most cases of gastric cancer in Western patients are diagnosed at an advanced stage, those with a locally advanced disease extending to the surrounding tissues (cT4b) may require a multivisceral resection (MVR), combining the stomach and adjacent organs, to provide the best chance of cure [3, 4].

The prevalence of combined resections for gastric cancer in various clinical registries reached between 10 and 30% with the pancreas, colon/mesocolon, and spleen among the most commonly resected organs [5–7]. However, the reported outcomes of MVRs remain highly inconsistent across studies with perioperative mortality and morbidity rates showing significant variability, ranging from 0 to 15% and 12 to 90%, respectively [4]. Part of the likely explanation is that usually all MVRs have been evaluated as a single entity without specifying results for individual organs [7–14]. Furthermore, most previous studies were underpowered, collecting data of relatively small populations not exceeding 100 cases [8, 14–22]. Consequently, still little is known about how the extent of a multivisceral resection necessary to achieve tumour clearance affects early and late postoperative outcomes.

The present study aimed to assess whether the postoperative course and survival of patients subject to MVRs for locally advanced gastric cancer (cT4b) were affected by the extent of organ resection. Moreover, we attempted to develop a nomogram estimating the risk of major postoperative morbidity after MVRs.

## Methods

### Patient population

An electronic database (Microsoft Access) of all patients with resectable gastric cancer treated between January 1996 and December 2020 in the First Department of Surgery, Jagiellonian University Medical College, an academic tertiary surgical centre, was reviewed. The extent of surgery, definitions for lymph node dissection, and tumour staging were adapted to the recent guidelines [2, 23]. Multivisceral resections (MVRs) were defined as partial or total gastrectomy combined with splenectomy, distal pancreatectomy, or partial colectomy for direct tumour infiltration suspected during surgery (cT4b). Pancreatic resections were always combined with splenectomy and were referred to as pancreatectomy. Patients who required resections of other organs and multiple organs were excluded to preserve the homogeneity of the study population. Ethical approval for the study was provided by the Bioethics Committee of the Jagiellonian University. The study has been registered in ClinicalTrials.gov (NCT01962519).

### Outcome measurements

Postoperative complications were defined as previously reported [24] and were classified according to the Clavien–Dindo scoring system [25]. Major postoperative complications were defined as grade 3a or higher. Postoperative mortality was defined as any death during the hospital stay after surgery. Overall survival was defined as the time from surgery to all-cause death or the date of the last follow-up. Dates of death were verified through the census registry office.

### Statistical analysis

All continuous variables are reported with their median and interquartile range (IQR) while categorical data are reported as proportions. Statistical significances of the differences in categorical and continuous variables were analysed by *χ*^2^ and the Mann–Whitney U tests where appropriate. The Bonferroni correction was used to account for multiple testing.

Potential predictors of major postoperative complications were initially evaluated by odds ratios (ORs) with 95% confidence intervals (CI). Three penalised regression methods (ridge, lasso, and elastic net regression) were used for the selection of predictive variables and formulation of a multivariable logistic regression model based on minimising the Akaike information criterion (AIC). Collinearity among variables was evaluated by variance inflation factors (VIF) with values greater than 5 used as a cut-off for multicollinearity. Model validation was performed by bootstrapping with 1000 resamples [26]. Calibration was evaluated with GiViTI calibration belt [27]. The predictive performance of the model was evaluated by the area under the receiver operating characteristic curve (AUC). A nomogram was formulated based on the results of the logistic regression model.

Survival data was analysed according to the Kaplan–Meier method and included postoperative mortality. Multivariate analysis was performed using a Cox proportional hazards model with a backward stepwise selection procedure. The significance level (*P*) < 0.05 in a two-tailed test was considered statistically significant. All statistical analyses were performed using the IBM® SPSS® Statistics 28 software package (IBM Corporation, NY) and RStudio (Integrated Development Environment for R) version 2021.9.2.382 with packages survival (3.3–1), rms (6.2–0), pROC (1.16.2), gtsummary (1.5.2), and caret (6.0–91).

## Results

### Patient characteristics

**Fig. 1:**
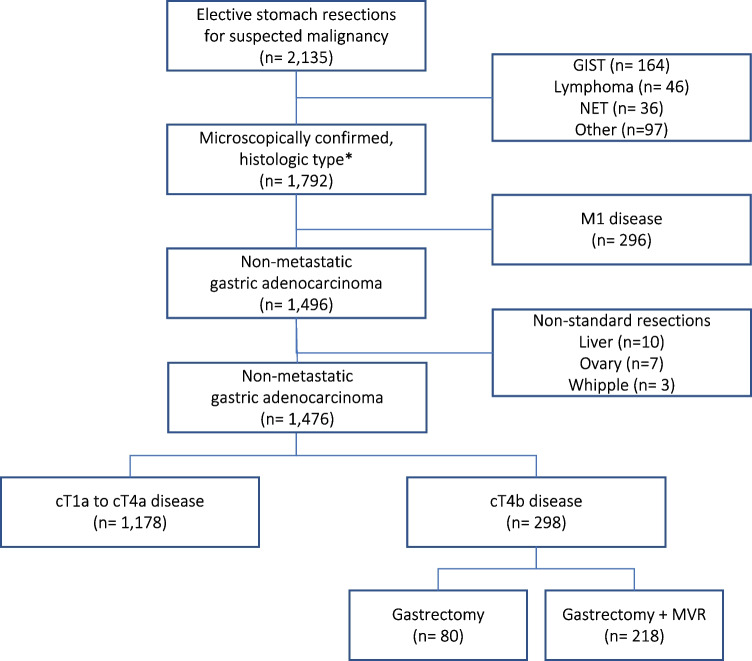
Flowchart of the study

A total of 1792 patients who underwent stomach resections for gastric cancer were identified between January 1996 and December 2020. Subsequently, 316 patients were excluded due to metastatic disease (*n* = 296) or combined resections of organs other than prespecified in inclusion criteria, including liver (*n* = 10), ovary (*n* = 7), and pancreatoduodenectomy (*n* = 3). Figure 1 shows the flowchart of the study. A group of 298 patients had locally advanced tumours with infiltration of surrounding organs suspected intraoperatively (cT4b disease), and they comprised the final study population. Approximately 62% of them were diagnosed preoperatively as locally advanced tumours. Preoperative chemotherapy was used in only 11% of patients, consisting mostly of three regimens, i.e. ECF (epirubicin, cisplatin, and fluorouracil), DCF (docetaxel, cisplatin, and 5-fluorouracil), and FLOT (5-fluorouracil, leucovorin, oxaliplatin, and docetaxel). Response rates to preoperative treatment were not routinely evaluated. Table 1 summarises the clinical data and patient characteristics between subjects with locally advanced disease and the remaining population. Two hundred and eighteen (73%) of 298 patients with cT4b tumours underwent MVRs, including the spleen (*n* = 126), pancreas (*n* = 51), and colon (*n* = 41, including right colectomy in 15 and partial resections in 26 patients). The overall proportion of patients with confirmed pT4b disease after MVR was 50% and was highest for colectomy (76%), followed by pancreatectomy (51%) and splenectomy (42%). The proportion was markedly higher among 80 subjects with cT4b disease who underwent gastrectomy alone and reached 97%.

**Table 1:** Patient demographics and clinicopathological parameters

Characteristic	Overall (*N* = 1476)	Clinical staging		*P*
		cT1a-cT4a (*N* = 1178)	cT4b (*N* = 298)	
Gender (male)	1017 (69%)	808 (69%)	209 (70%)	0.607^†^
Age (≥ 65 y)	775 (53%)	616 (52%)	159 (53%)	0.743^†^
Race/ethnicity (Caucasian)	1476 (100%)	1178 (100%)	298 (100%)	1.000^†^
Comorbidities				
Arterial hypertension	659 (45%)	540 (46%)	119 (40%)	0.067^†^
Ischemic heart disease	401 (27%)	327 (28%)	74 (25%)	0.310^†^
Arrhythmia	142 (9.6%)	122 (10%)	20 (6.7%)	0.057^†^
Chronic heart failure	60 (4.1%)	55 (4.7%)	5 (1.7%)	0.019^†^
COPD/asthma	122 (8.3%)	101 (8.6%)	21 (7.0%)	0.392^†^
Diabetes	227 (15%)	183 (16%)	44 (15%)	0.742^†^
Liver cirrhosis	26 (1.8%)	21 (1.8%)	5 (1.7%)	0.902^†^
CKD	31 (2.1%)	29 (2.5%)	2 (0.7%)	0.054^†^
ASA class (3 or 4)	346 (23%)	285 (24%)	61 (20%)	0.175^†^
Charlson comorbidity index				0.023^†^
0	847 (57%)	662 (56%)	185 (62%)	
1	437 (30%)	349 (30%)	88 (30%)	
≥ 2	192 (13%)	167 (14%)	25 (8.4%)	
Weight loss	876 (59%)	658 (56%)	218 (73%)	< 0.001^†^
Percent weight loss^*^	11 (7, 16)	10 (7, 15)	14 (9, 20)	< 0.001^‡^
Body mass index^*^	24.7 (22.1, 27.7)	24.9 (22.3, 27.8)	23.9 (21.6, 26.8)	< 0.001^‡^
Tumour location (distal)	537 (36%)	462 (39%)	75 (25%)	< 0.001^†^
Tumour size (mm)^*^	50 (30, 80)	50 (30, 70)	90 (60, 115)	< 0.001^‡^
Lauren type, intestinal	741 (50%)	626 (53%)	115 (39%)	< 0.001^†^
Tumour grade (2 or 3)	1,274 (87%)	995 (85%)	279 (94%)	< 0.001^†^
Lymphovascular invasion	515 (35%)	383 (33%)	132 (44%)	< 0.001^†^
Perineural invasion	255 (17%)	191 (16%)	64 (21%)	0.032^†^
Neoadjuvant chemotherapy	156 (11%)	125 (11%)	31 (10%)	0.917^†^
Gastrectomy (total)	943 (64%)	689 (58%)	254 (85%)	< 0.001^†^
Lymphadenectomy D2	1,066 (72%)	843 (72%)	223 (75%)	0.260^†^
Surgeon caseload (≥ 100)	1,174 (80%)	937 (80%)	237 (80%)	0.997^†^
Organ resection	432 (29%)	214 (18%)	218 (73%)	< 0.001^†^
Spleen	340 (23%)	214 (18%)	126 (42%)	< 0.001^†^
Pancreas	51 (3.5%)	0 (0%)	51 (17%)	< 0.001^†^
Colon	41 (2.8%)	0 (0%)	41 (14%)	< 0.001^†^
Curative resection (R0)	1220 (83%)	1082 (92%)	138 (46%)	< 0.001^†^
Blood transfusion (yes)	476 (32%)	342 (29%)	134 (45%)	< 0.001^†^
Blood transfusion (ml)^*^	440 (410, 830)	440 (300, 660)	545 (440, 880)	< 0.001^‡^
Resected lymph nodes^*^	24 (15, 32)	23 (14, 31)	26 (18, 34)	< 0.001^‡^
Metastatic lymph nodes^*^	3 (0, 12)	2 (0, 9)	11 (3, 21)	< 0.001^‡^
Depth of infiltration (pT)				< 0.001^†^
T1a	112 (7.6%)	112 (9.5%)	0 (0%)	
T1b	130 (8.8%)	130 (11%)	0 (0%)	
T2	150 (10%)	150 (13%)	0 (0%)	
T3	653 (44%)	637 (54%)	16 (5.4%)	
T4a	243 (16%)	149 (13%)	94 (32%)	
T4b	188 (13%)	0 (0%)	188 (63%)	
Lymph node status (pN)				< 0.001^†^
N0	512 (35%)	474 (40%)	38 (13%)	
N1	191 (13%)	158 (13%)	33 (11%)	
N2	199 (13%)	157 (13%)	42 (14%)	
N3a	297 (20%)	226 (19%)	71 (24%)	
N3b	277 (19%)	163 (14%)	114 (38%)	

^*^Median (interquartile range)

^†^Pearson’s chi-squared test

^‡^Mann–Whitney U test

*ASA*, American Society of Anesthesiologists; *CKD*, chronic kidney disease; *COPD*, chronic obstructive pulmonary disease; *BMI*, body mass index

### Postoperative outcomes for locally advanced (cT4b) tumours

The overall postoperative morbidity was 75%. Grades I and II complications, according to the Clavien–Dindo classification, were found in 44% of patients, and more than one half of them (24%) were due to red blood cell transfusions associated with a more liberal transfusion strategy in the early study period. Compared to gastric resections alone, MVRs were associated with increased postoperative morbidity (80% vs 62%, *P* = 0.002) caused by higher proportions of surgical (41% vs 29%, *P* = 0.048) and general complications (75% vs 60%, *P* = 0.013). Patients after MVRs had higher median values of the Comprehensive Complication Index (CCI 26 vs 21, *P* = 0.006), and longer median duration of hospital stay (13 vs 8 days, *P* < 0.001). However, the increased rates of grade ≥ 3a complications (34% vs 22%, *P* = 0.058), surgical reinterventions (17% vs 11%, *P* = 0.261), and hospital readmissions (9.2% vs 5.0%, *P* = 0.241) were insignificant. The overall mortality rate of 5.0% was equal for gastrectomy alone and MVRs.

### Organ-specific morbidity for MVRs for cT4b tumours

**Fig. 2:**
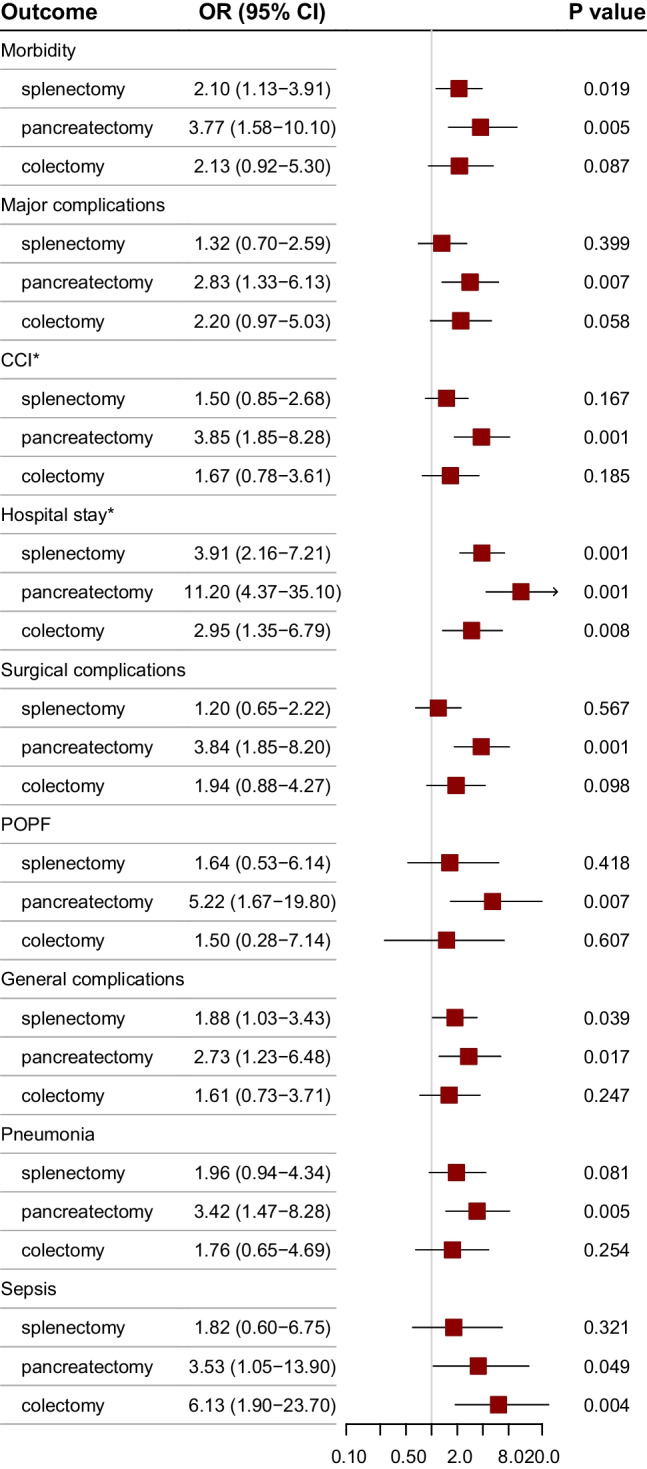
Odds ratios (OR, 95% CI) of splenectomy, pancreatectomy, and colectomy compared to gastrectomy alone for postoperative outcome measures identified as possibly related to MVRs. *OR for the likelihood of achieving values higher than the median value for gastrectomy alone

Postoperative outcomes stratified by the resected organ are summarised in supplementary table 1. Figure 2 shows odds ratios of splenectomy, pancreatectomy, and colectomy compared to gastrectomy alone for outcome measures identified as possibly related to MVRs. Pairwise comparisons demonstrated that pancreatectomy compared to gastrectomy alone was associated with higher overall (*P* = 0.005), surgical (*P* = 0.001), general (*P* = 0.017), and major (*P* = 0.007) complications, as well as pancreatic fistula (*P* = 0.007), pneumonia (*P* = 0.005), and sepsis (*P* = 0.049). Distal pancreatectomy also increased the odds for achieving higher values of CCI (*P* = 0.001) and longer hospital stay (*P* = 0.001). Splenectomy was associated with higher odds for overall morbidity (*P* = 0.019), general complications (*P* = 0.039), and prolonged hospital stay (*P* = 0.001) but had no effect on individual complications. On the other hand, colonic resections significantly increased the odds of septic complications (*P* = 0.004) and prolonged hospital stay (*P* = 0.008) compared to gastrectomy alone.

### Prediction of major postoperative complications for cT4b tumours

**Fig. 3:**
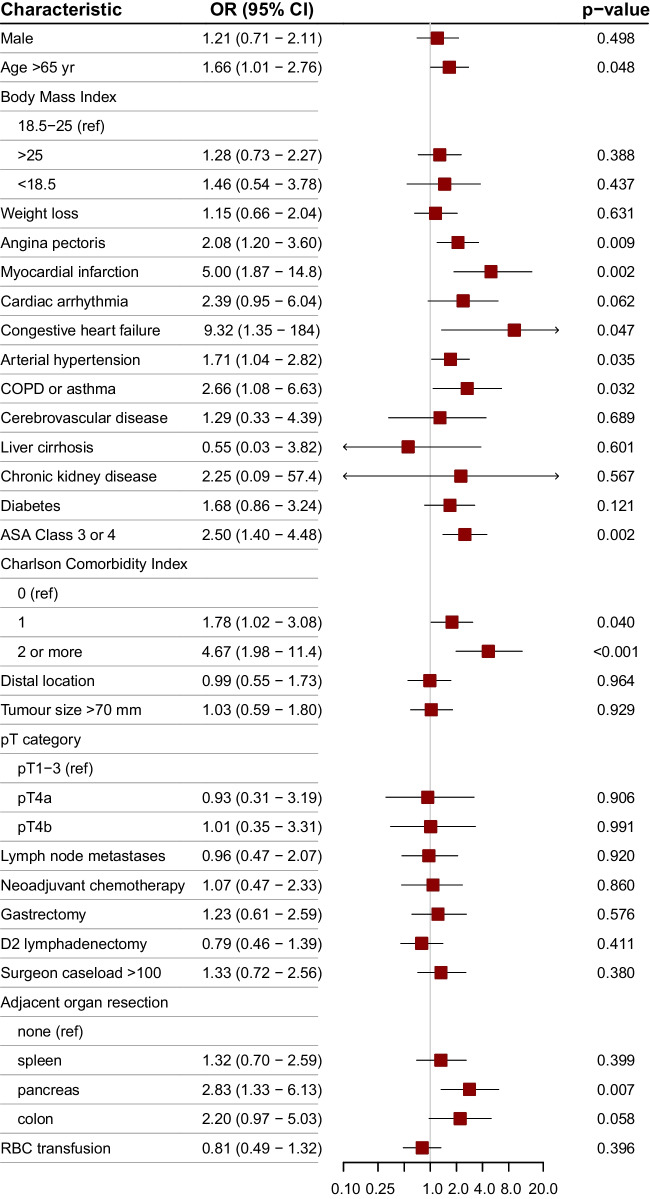
Univariate logistic analysis of potential predictors for major postoperative morbidity (Clavien–Dindo ≥ 3a) among patients with cT4b disease. ASA, American Society of Anesthesiologists; COPD, chronic obstructive pulmonary disease; RBC, red blood cell

**Fig. 4:**
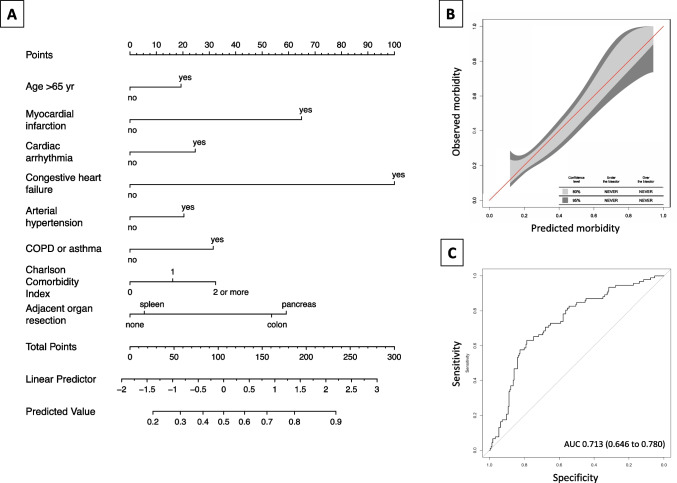
Results of a logistic regression model predicting major postoperative morbidity (Clavien–Dindo ≥ 3 a). **A** The nomogram is used by adding up the points identified on the points scale for each variable. The total points projected on the bottom scale indicate the probability of major morbidity. **B** Model calibration using GiViTI calibration belt. **C** Receiver operating characteristic plot for the model

Figure 3 shows the results of univariate logistic analysis of potential predictors for complications grade 3a or higher according to the Clavien–Dindo classification. Subsequently, penalised regression analysis was employed to build a multivariable logistic regression model predicting major postoperative morbidity. The best goodness of fit, as shown by the lowest AIC, was found for the model including 8 variables, i.e. age > 65 years, history of myocardial infarction, cardiac arrhythmia, congestive heart failure, arterial hypertension, COPD or asthma, Charlson’s comorbidity index, and multivisceral resection. Although splenectomy was not associated with major morbidity, distal pancreatectomy and colectomy were identified as independent risk factors with odds ratios of 2.53 (95% CI 1.23–5.19, *P* = 0.012) and 2.29 (95% CI 1.04–5.09, *P* = 0.035), respectively. Model calibration by bootstrapping (mean absolute error of 0.01) and GiViTi calibration belt showed very good correlation between the predicted and actual probability of major postoperative complications. The AUC of ROC obtained from the model was 0.713 (95% CI 0.646 to 0.780). A nomogram to calculate the probability of major postoperative complications is presented in Fig. 4. History of congestive heart failure or myocardial infarction, as well as distal pancreatectomy and colectomy, had the greatest impact on grade ≥ 3a complications.

### Prognosis of locally advanced (cT4b) tumours

**Fig. 5:**
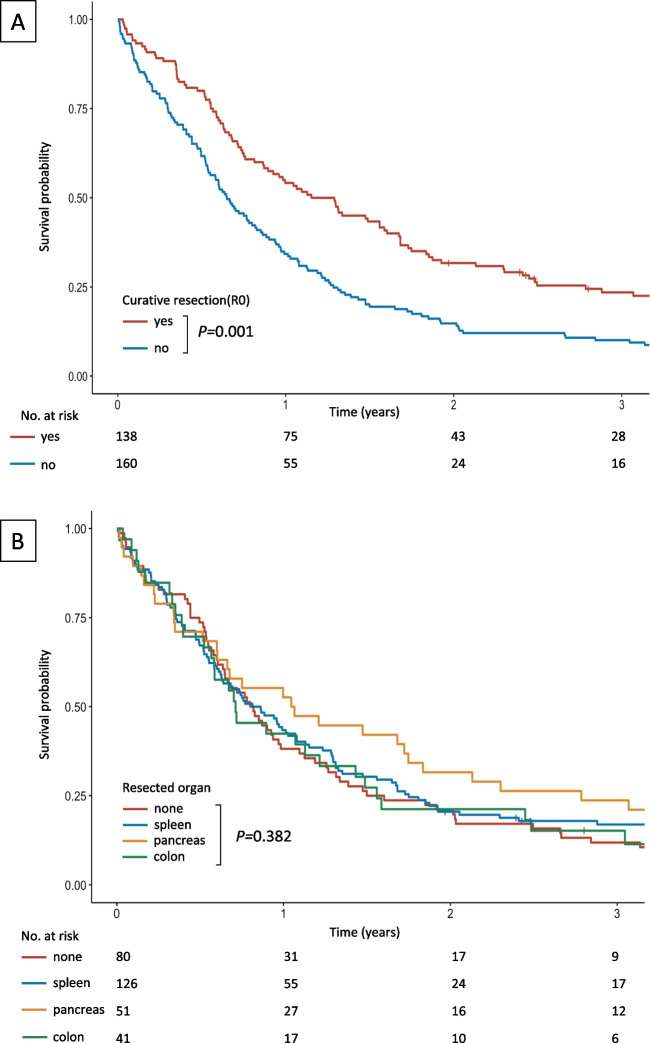
Kaplan–Meier curves for overall survival of patients who underwent surgery for cT4b gastric cancer for groups defined by curability of resection (**A**) and the extent of resection (**B**) (log-rank test)

At the time of final follow-up, 1059 of 1476 patients (72%) had died. The median follow-up for all surviving subjects was 101 months, and 151 months for patients in the cT4b group. The overall median survival for the latter group was 10.6 months (95% CI 8.7–12.5) and was significantly longer after microscopically radical (R0) resections (15.8 vs 7.9 months, *P* < 0.001, Fig. 5). The overall median survival (95% CI) was comparable (*P* = 0.382) for patients without MVRs (9.7 months, 7.3–12.2) and after resection of the spleen (9.9 months, 7.2–12.6), pancreas (12.7 months, 4.3–21.2), and colon (8.7 months, 4.4–12.9). A Cox proportional hazards model (Table 2) identified non-radical resection as the most important risk factor for patients’ survival (HR 1.47), followed by age above 65 years (HR 1.43), and non-intestinal histology (HR 1.25). In the MVR group, the overall median survival of patients with pT3/T4a and pT4b tumours was 11.7 and 8.5 months (*P* = 0.042), respectively. The median survival was also significantly affected by lymph node status and was markedly shorter in patients with positive lymph nodes (10.7 vs 22.5 months, *P* < 0.001).

**Table 2:** Univariate and multivariate analyses of prognostic factors in patients with locally advanced (cT4b) tumours (*n* = 298)

Characteristic	Univariate		Multivariate	
	HR (95% CI)	*P*-value	HR (95% CI)	*P*-value
Gender (male)	1.16 (0.88–1.53)	0.280		
Age (≥ 65 y)	1.53 (1.20–1.96)	< 0.001	1.43 (1.10–1.87)	0.008
Weight loss	1.51 (1.14–2.01)	0.004	1.16 (0.85–1.59)	0.346
ASA class (3 or 4)	1.37 (1.01–1.86)	0.041	1.21 (0.88–1.68)	0.241
Tumour location (distal)	1.08 (0.82–1.43)	0.573		
Tumour size (> 70 mm)	1.37 (1.04–1.82)	0.026	1.20 (0.89–1.63)	0.229
Lauren type (intestinal)	0.74 (0.58–0.96)	0.021	0.75 (0.57–0.99)	0.039
Tumour grade (2 or 3)	1.54 (0.91–2.60)	0.104	0.96 (0.54–1.72)	0.888
Lymphovascular invasion	1.30 (1.02–1.67)	0.035	1.13 (0.87–1.47)	0.355
Perineural invasion	0.99 (0.73–1.35)	0.954		
Neoadjuvant chemotherapy	0.64 (0.41–0.99)	0.047	0.71 (0.45–1.12)	0.145
RBC transfusion	0.95 (0.74–1.21)	0.672		
Gastrectomy, total	1.13 (0.80–1.58)	0.492		
Lymphadenectomy D2	1.03 (0.78–1.37)	0.824		
Surgeon caseload (> 100)	0.81 (0.60–1.09)	0.159		
Multivisceral resection	0.77 (0.59–1.01)	0.059	0.94 (0.68–1.29)	0.699
Non-radical resection (R1)	1.78 (1.39–2.29)	< 0.001	1.47 (1.10–1.97)	0.010
Depth of infiltration (pT)				
T1a–T3	Reference		Reference	
T4a	2.24 (1.15–4.34)	0.017	1.55 (0.78–3.10)	0.210
T4b	2.80 (1.47–5.34)	0.002	1.74 (0.87–3.49)	0.120
Lymph node status (pN)				
N0	Reference		Reference	
N1 or N2	1.34 (0.87–2.07)	0.184	1.19 (0.75–1.88)	0.459
N3a or N3b	1.84 (1.25–2.72)	0.002	1.50 (0.98–2.28)	0.062

*HR*, hazard ratio; *CI*, confidence intervals; *RBC*, red blood cell

Margin-negative (R0) resections were carried out in 138 (46%) of 298 patients with cT4b disease. Non-radical resections were due to infiltration of transection margin (*n* = 33, 11%), radial margin (*n* = 87, 30%), and both margins (*n* = 40, 13%). The proportion of margin-positive resections was highest after splenectomy (56%), followed by colectomy (34%), and pancreatectomy (22%). A logistic regression analysis was carried out to identify factors associated with the risk of positive resection margins among patients with cT4b disease (Supplementary Table 2). The odds of non-radical resection were significantly higher for moderately/poorly differentiated tumours (OR 6.52, 95% CI 1.86–31.0, *P* = 0.007) and for pathologically confirmed infiltration of surrounding organs (OR 10.2, 95% CI 2.61–67.6, *P* = 0.003). Multivisceral resection was the only factor reducing the risk of margin positivity (OR 0.44, 95% CI 0.21–0.87, *P* = 0.022).

## Discussion

Locally advanced gastric cancer requiring multivisceral resections of surrounding organs continues to pose an important clinical challenge. This single institutional study using data from a prospectively maintained database of Western patients found that MVRs significantly increased the odds of achieving complete (R0) tumour clearance. However, such resections were associated with increased postoperative morbidity related to the type of the organ being resected.

About 20–30% pathology reports for resected gastric cancers demonstrate tumour invasion of either serosal membrane (pT4a) or adjacent structures (pT4b) [28, 29]. In the latter case, the pancreas, the spleen, the transverse colon, and the liver are most commonly affected, determining the ability to achieve potentially curative resections [12, 22, 30]. Desmoplastic and inflammatory reactions around pT3 and pT4a tumours may sometimes mimic true invasion of surrounding organs (pT4b) and 20–60% of pathology reports fail to confirm the T4b disease [7, 8, 18–21, 31]. Therefore, the decision about the need to perform a MVR is made intraoperatively without knowing the exact extent of the disease, and one must balance the oncological benefits against the potential risks from extended resections.

The scepticism regarding MVRs in gastric cancer was primarily driven by an increased risk of postoperative morbidity reported in most studies [4]. Resection of additional organs was generally accompanied by higher rates of major postoperative complications (Clavien–Dindo ≥ 3) in about 30% of cases [7, 32] and mortality of up to 9–14% [7–9]. However, detailed information concerning early postoperative period in patients subject to MVRs for locally advanced cancers is generally missing and little is known about the risk associated with the resection of individual organs based on previous reports [10, 31, 33–36].

In the present study, MVRs among patients with locally advanced tumours were associated with an increased overall morbidity as well as higher proportions of surgical and general complications. An explanatory analysis demonstrated that these effects were mainly due to morbidity attributed to pancreatic and colonic resections, but not splenectomy. Both types of resections significantly increased odds ratios for major complications reaching 2.53 for distal pancreatectomy and 2.29 for colectomy, but the mortality rates were unaffected. Interestingly, the type of the resected organ was the only surgery-related parameter included in the nomogram developed to predict major complications. Intuitively, one could anticipate increased morbidity after MVRs based on data obtained from randomised clinical trials evaluating planned pancreatectomy and splenectomy upon initial experience with D2 lymphadenectomy [37]. However, such findings were rarely reported for resections carried out for locally advanced tumours. Similarly to our results, Min et al. in a population of 243 patients with locally advanced cancers (pT4b) demonstrated morbidity rates higher for pancreatic resections (30%) than for partial colectomy (13%) or hepatectomy (19%) [38]. However, some contrary results were reported by van der Werf et al. who demonstrated similar rates of Clavien–Dindo grade 3 or higher complications among patients with pT4 tumours after partial pancreatectomy (17%) and gastrectomy alone (17%) [39].

Previous studies established that the completeness of resection, achievable in about 30–85% of cases, was the most important predictor of long-term survival after MVRs for gastric cancer [8, 10, 12, 19, 21, 30–33, 35]. Most reports also failed to demonstrate significant prognostic differences among patients subject to MVRs and related to the type of the resected organ, including the spleen, pancreas, or colon [10, 12, 18, 32, 33]. Even though the resected organ does not seem to determine survival, as in our cohort, some data suggest that it may be related to the likelihood of curative resections with the highest rates reported among patients requiring pancreaticosplenectomy [7, 19, 35]. This was somehow conflicting with findings suggesting significantly poorer survival after pancreatic resection. Among 243 patients with pT4b disease, Min et al. found that only patients with pancreatic invasion (*n* = 67) had higher hazard ratio for death (1.46, 95% CI 1.01–2.11, *P* = 0.043), and no such differences were demonstrated for tumours invading the colon or liver [38]. Similar findings were reported in a group where pancreatectomy was associated with increased hazard ratio for death (1.67, 95% CI 1.02–2.76, *P* = 0.044) [7].

Our results provide detailed data from a Western population supporting safety and long-term outcomes of MVRs for gastric cancer. However, two important limitations should also be considered. First, due to the retrospective nature of this study, we could not strictly define the reasons that prompted the decision to perform resection of adjacent organs or gastrectomy alone for cT4b tumours. Generally, the decision was left to the surgeon and was balanced between the feasibility of performing a macroscopically curative resection and the overall perioperative risk for the patient. Second, the proportion of patients with locally advanced cancers receiving perioperative chemotherapy was low considering the current guidelines recommending perioperative treatment for most patients. As chemotherapy prior to surgery lowered the HR of death in the univariate analysis, a more common use of systemic treatment could likely increase the rates of R0 resections and further improve long-term results.

In conclusion, the present study demonstrated that MVRs for locally advanced gastric cancer significantly increased the likelihood of achieving tumour-free resection margins. Resection of organs adjacent to the stomach increased the risk of postoperative complications and prolonged hospital stay in an organ-dependent manner. Analysis of further large-scale population-based data sets is still desirable to determine whether preoperative treatment may further potentiate the benefits from MVRs.

### Supplementary Information

Below is the link to the electronic supplementary material.

Supplementary file1 (DOCX 25 KB)

## Data Availability

The data that support the findings of this study are available from the authors upon reasonable request.
