# Supplementary material for: What determines complications and prognosis among patients subject to multivisceral resections for locally advanced gastric cancer?
Source: Langenbecks Arch Surg. 2023 Nov 21;408(1):442. doi: 10.1007/s00423-023-03187-7 (PMC10663187; doi:10.1007/s00423-023-03187-7)
Supplement: Supplementary file 1 — Supplementary file1 (DOCX 25 KB) [file 423_2023_3187_MOESM1_ESM.docx]

Supplementary Table 1. Postoperative outcomes by the type of multivisceral resection (MVR) in patients with locally advanced (cT4b) tumours (n=298)

| **Characteristic** | **Overall (N= 298)** | **Type of MVR** | | | | ***P*** |
| --- | --- | --- | --- | --- | --- | --- |
|  |  | none (N=80) | spleen (N=126) | pancreas (N=51) | colon (N=41) |  |
| Morbidity | 224 (75%) | 50 (62%) | 98 (78%) | 44 (86%) | 32 (78%) | 0.012^†^ |
| Clavien-Dindo grade 1-2 | 132 (44%) | 32 (40%) | 63 (50%) | 21 (41%) | 16 (40%) | 0.404^†^ |
| Clavien-Dindo grade ≥3a | 92 (31%) | 18 (22%) | 35 (28%) | 23 (45%) | 16 (39%) | 0.025^†^ |
| CCI^*^ | 21 (9, 43) | 21 (0, 30) | 21 (21, 39) | 31 (21, 52) | 21 (21, 52) | 0.004^‡^ |
| Mortality, in hospital | 15 (5.0%) | 4 (5.0%) | 7 (5.6%) | 3 (5.9%) | 1 (2.4%) | 0.926^†^ |
| **Mortality, 90 days** | **31 (10%)** | **7 (8.8%)** | **12 (9.5%)** | **8 (16%)** | **4 (9.8%)** | **0.616** |
| Reoperation | 45 (15%) | 9 (11%) | 16 (13%) | 12 (24%) | 8 (20%) | 0.176^†^ |
| Hospital stay, days^*^ | 12 (8, 23) | 8 (7, 17) | 13 (9, 21) | 18 (10, 36) | 13 (7, 25) | <0.001^‡^ |
| Readmission | 24 (8.1%) | 4 (5.0%) | 11 (8.7%) | 5 (9.8%) | 4 (9.8%) | 0.639^†^ |
| *Surgical complications* | 113 (38%) | 23 (29%) | 41 (33%) | 31 (61%) | 18 (44%) | <0.001^†^ |
| anastomotic leak | 18 (6.0%) | 3 (3.8%) | 8 (6.3%) | 4 (7.8%) | 3 (7.3%) | 0.724^†^ |
| abscess | 24 (8.1%) | 4 (5.0%) | 8 (6.3%) | 6 (12%) | 6 (15%) | 0.173^†^ |
| fluid collection | 21 (7.0%) | 4 (5.0%) | 6 (4.8%) | 6 (12%) | 5 (12%) | 0.158^†^ |
| ileus | 12 (4.0%) | 3 (3.8%) | 4 (3.2%) | 3 (5.9%) | 2 (4.9%) | 0.785^†^ |
| abdominal bleeding | 9 (3.0%) | 2 (2.5%) | 3 (2.4%) | 2 (3.9%) | 2 (4.9%) | 0.781^†^ |
| wound complications | 28 (9.4%) | 7 (8.8%) | 8 (6.3%) | 8 (16%) | 5 (12%) | 0.224^†^ |
| POPF | 28 (9.4%) | 4 (5.0%) | 10 (7.9%) | 11 (22%) | 3 (7.3%) | 0.022^†^ |
| other | 6 (2.0%) | 1 (1.2%) | 3 (2.4%) | 0 (0%) | 2 (4.9%) | 0.418^†^ |
| *General complications* | 211 (71%) | 48 (60%) | 93 (74%) | 41 (80%) | 29 (71%) | 0.062^†^ |
| pneumonia | 68 (23%) | 11 (14%) | 30 (24%) | 18 (35%) | 9 (22%) | 0.040^†^ |
| urinary tract infection | 2 (0.7%) | 2 (2.5%) | 0 (0%) | 0 (0%) | 0 (0%) | 0.166^†^ |
| circulatory | 53 (18%) | 12 (15%) | 21 (17%) | 12 (24%) | 8 (20%) | 0.622^†^ |
| respiratory | 38 (13%) | 8 (10%) | 19 (15%) | 7 (14%) | 4 (9.8%) | 0.675^†^ |
| hepatic failure | 3 (1.0%) | 0 (0%) | 3 (2.4%) | 0 (0%) | 0 (0%) | 0.460^†^ |
| renal failure | 7 (2.3%) | 1 (1.2%) | 4 (3.2%) | 1 (2.0%) | 1 (2.4%) | 0.944^†^ |
| sepsis | 33 (11%) | 4 (5.0%) | 11 (8.7%) | 8 (16%) | 10 (24%) | 0.008^†^ |
| PE/DVT | 4 (1.3%) | 1 (1.2%) | 3 (2.4%) | 0 (0%) | 0 (0%) | 0.900^†^ |
| other^§^ | 140 (47%) | 36 (45%) | 61 (48%) | 24 (47%) | 19 (46%) | 0.971^†^ |

CCI, Comprehensive Complication Index; POPF, Postoperative pancreatic fistula

^*^Median (interquartile range); ^†^ Pearson's Chi-squared test; ^‡^ Kruskal-Wallis test

^§^ including postoperative RBC transfusions

Supplementary Table 2. Univariate and multivariate regression analysis of risk factors for margin-positive resections in patients with locally advanced (cT4b) tumours (n=298)

| **Characteristic** | **Univariate** | | **Multivariate** | |
| --- | --- | --- | --- | --- |
|  | **OR (95% CI)** | **p-value** | **OR (95% CI)** | **p-value** |
| Gender, male | 0.67 (0.40 – 1.10) | 0.116 |  |  |
| Age, ≥65 y | 1.29 (0.81 – 2.03) | 0.281 |  |  |
| Body Mass Index |  |  |  |  |
| 18.5–25 | reference |  |  |  |
| >25 | 1.18 (0.69 – 2.02) | 0.539 |  |  |
| <18.5 | 2.16 (0.82 – 6.40) | 0.134 |  |  |
| Tumour location, distal | 1.52 (0.89 – 2.61) | 0.126 |  |  |
| Tumour size, >70 mm | 0.94 (0.56 – 1.56) | 0.800 |  |  |
| Lauren type, intestinal | 0.72 (0.45 – 1.15) | 0.168 |  |  |
| Tumour grade, 2 or 3 | 5.92 (1.88 – 26.1) | 0.006 | 6.52 (1.86 – 31.0) | 0.007 |
| Lymphovascular invasion | 1.01 (0.64 – 1.59) | 0.976 |  |  |
| Perineural invasion | 1.05 (0.60 – 1.84) | 0.857 |  |  |
| Neoadjuvant chemotherapy | 0.51 (0.23 – 1.08) | 0.081 | 0.78 (0.32 – 1.89) | 0.589 |
| Gastrectomy, total | 0.77 (0.40 – 1.47) | 0.437 |  |  |
| Lymphadenectomy D2 | 0.38 (0.21 – 0.66) | <0.001 | 0.65 (0.33 – 1.26) | 0.207 |
| Surgeon caseload >100 | 0.76 (0.43 – 1.34) | 0.350 |  |  |
| Multivisceral resection | 0.20 (0.10 – 0.35) | <0.001 | 0.44 (0.21 – 0.87) | 0.022 |
| Depth of infiltration, pT |  |  |  |  |
| T1a–T3 | reference |  | reference |  |
| T4a | 2.97 (0.76 – 19.7) | 0.168 | 2.68 (0.67 – 18.0) | 0.215 |
| T4b | 15.7 (4.21 – 102) | <0.001 | 10.2 (2.61 – 67.6) | 0.003 |
| Lymph node status, pN |  |  |  |  |
| N0 | reference |  |  |  |
| N1 or N2 | 1.41 (0.65 – 3.12) | 0.389 |  |  |
| N3a or N3b | 1.55 (0.77 – 3.16) | 0.220 |  |  |

OR, odds ratio; CI, confidence intervals
